# Zinc‐induced copper deficiency, sideroblastic anemia, and neutropenia: A perplexing facet of zinc excess

**DOI:** 10.1002/ccr3.2987

**Published:** 2020-05-28

**Authors:** Ahsan Wahab, Kamran Mushtaq, Samuel G. Borak, Naresh Bellam

**Affiliations:** ^1^ Baptist Medical Center South University of Alabama at Birmingham Montgomery AL USA; ^2^ Northeast Internal Medicine Associates LaGrange IN USA; ^3^ UAB Department of Pathology Baptist Health Montgomery AL USA; ^4^ Montgomery Cancer Center Prattville AL USA

**Keywords:** copper, neutropenia, sideroblastic anemia, zinc

## Abstract

Hypocupremia due to zinc products can cause sideroblastic anemia and neutropenia and mimics other serious hematological disorders. Early consideration of the copper deficiency and a thorough clinical history can prevent unnecessary interventions.

## Graphical abstract

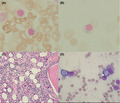

## 1 INTRODUCTION

Hyperzincemia‐induced hypocupremia can cause sideroblastic anemia, leukopenia, and neutropenia. Therefore, it is important to remember hypocupremia as a potential etiology in patients presenting with cytopenias. Earlier detection of hypocupremia avoids future morbidity of neurological deficits, a decline in quality of life due to cytopenias, and avoids continued ineffective interventions.

Hypomineralinemia such as hypocupremia, a nutritional deficiency, is reported to cause a multitude of problems, including both hematologic and neurologic.^1^, ^2^ Hyperzincemia‐induced hypocupremia has also been outlined in the literature.^3^, ^4^, ^5^ Hyperzincemia, the disorder of zinc excess, occurs due to the ingestion of zinc supplements or the application of zinc products.^6^, ^7^, ^8^ Prolonged and excessive exposure to zinc leads to reduced copper absorption from the gastrointestinal tract and cause hypocupremia. Copper is a cofactor or coenzyme of many essential human enzymes such as superoxide dismutase, cytochrome‐c oxidase, and ceruloplasmin. Deficiency of copper can occur in several conditions such as reduced oral intake, impaired absorption, or excessive urinary and gastrointestinal losses of copper (Table 1: Major Causes of Sideroblastic Anemia, Nutritional Disorders).^9^

**TABLE 1:** Major causes of sideroblastic anemia

Main classes	Subclasses
Neoplastic disorders	MDS with RS with single or multilineage dysplasia. Myelodysplastic/Myeloproliferative disorder with RS and thrombocytosis.^17^
Nutritional disorders	Zinc excess.^3^, ^4^, ^5^, ^6^, ^7^, ^8^ Copper deficiency (reduced intake,^18^ malabsorption^19^/Celiac disease,^1^ small bowel disorder or surgeries,^1^ artificial nutritional feeding such as PEG tube feeds,^20^ TPN,^21^ colloidal silver infusions,^22^ and increased urinary loss.^23^
Toxins/Drugs	Excessive alcohol intake.^24^ Drugs (isoniazid and chloramphenicol).^25^ Others.
Inherited disorders	X‐linked sideroblastic anemia with/without ataxia.^25^ Mitochondrial disorders. Syndromic disorders.

Excessive zinc administration in the form of topical, oral, or enteral products, including but not limited to acne creams, wound care products, OTC zinc‐related supplements, denture adhesive creams, and ingestion of metallic objects, is attributed to causing hypocupremia.^3^, ^4^, ^5^, ^6^, ^7^, ^8^, ^10^

Hypocupremia‐induced hematologic abnormalities are often misdiagnosed as other disorders.^11^ Hematologically, copper depletion can present as anemia with or without leukopenia, with/without thrombocytopenia, pancytopenia, or isolated neutropenia.^1^ Sideroblastic anemia with neutropenia due to hypocupremia has also been described.^3^, ^4^ We present a case of a 30‐year‐old woman who developed hyperzincemia due to zinc‐containing denture adhesive creams, developed hypocupremia, and presented with symptoms of severe anemia; she was later diagnosed with sideroblastic anemia and neutropenia.

## 2 CASE REPORT

A thirty‐year‐old Caucasian nonalcoholic woman came to the ER with complaints of dizziness, lightheadedness, and palpitations. She reported no loss of consciousness. She denied a history or active complaints of heavy menstrual and gastrointestinal bleeding. She reported no fever, night sweats, weight loss, rash, and viral syndrome. Upon arrival, her vitals were stable (blood pressure: 126/77 mmHg, pulse: 107/minute (sinus), respiratory rate: 24/minute, oxygen saturation: 98% on room air). On physical examination, she looked pale without signs of distress. Oral mucosa was moist. There was no cervical lymphadenopathy. Lungs were clear to auscultate. Cardiovascular auscultation revealed sinus tachycardia without added sounds. Her abdomen was soft and nontender without palpable masses. Laboratories performed in the ER showed WBC 2.7 × 10^−3^/μL (4.1‐10.3), hemoglobin 4.5 gm/dL (11.3‐15.3), hematocrit 16.1% (34‐46), MCV 91 FL (81‐100), MCHC 28 gm/dL (32‐35), platelet count 256 × 10^3^/μL (140‐400), and RDW 19.4% (11.5‐14.5). Absolute neutrophil count was 1.3 × 10^3^/μL while other white cell counts were within normal limits. Comprehensive chemistry panel was unremarkable. β‐HCG was negative. Given severe symptomatic anemia, she was given two units of packed red blood cells, and she was admitted for anemia and neutropenia workup. Iron studies showed iron deficiency (serum ferritin 47.7 ng/mL (10‐200), iron saturation 8% (20‐55), TIBC 500 μg/dL (250‐460), iron 41.0 μg/dL (50‐170)). Stool occult blood was negative. Serum folate level was 12.9 ng/mL (>3.1) and vitamin B12 level 246 pg/mL (193‐986). Serum LDH was 145 ng/mL (84‐246). The patient's reticulocyte count was inappropriately low, consistent with ineffective erythropoiesis.

Examination of the peripheral blood smear demonstrated normocytic, normochromic erythrocytes with mild anisopoikilocytosis, including occasional elliptocytes. White blood cells were decreased in number and consisted predominantly of mature lymphocytes; blasts were not identified. The remaining white blood cells consisted of morphologically unremarkable neutrophils and monocytes. There was no overt evidence of dysplasia. Platelets were within the normal range. CT of the abdomen and pelvis without contrast to rule out other causes showed an enlarged right lobe of the liver, measuring 20 cm. Spleen was also mildly enlarged, measuring 13.4 × 14.7 cm. No lymphadenopathy was seen. CT chest with contrast also showed no lymphadenopathy.

**FIGURE 1:**
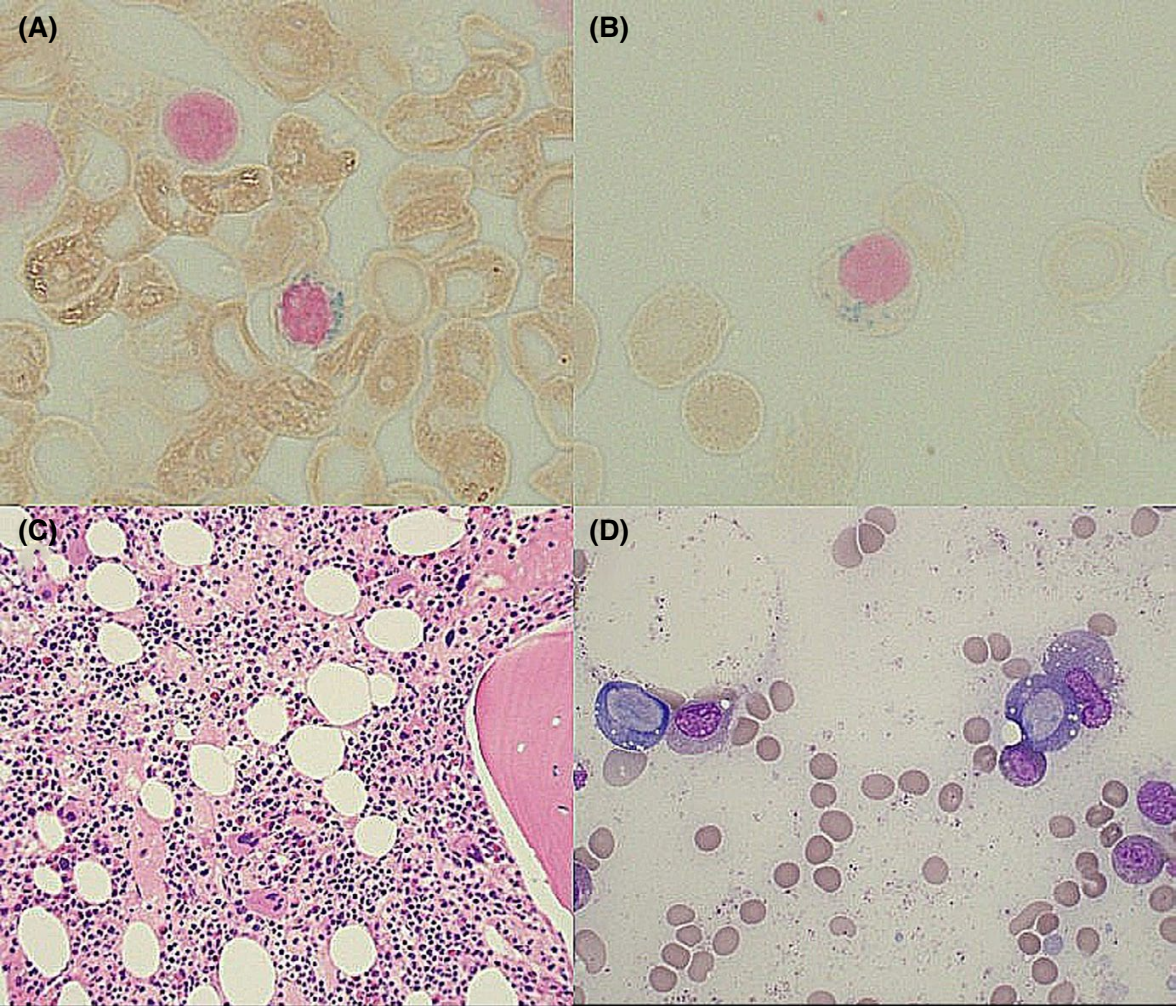
Bone marrow biopsy histopathology findings showing (A) ring sideroblast, (B) iron granules in plasma cell, (C) core biopsy with hypercellular marrow, and (D) vacuoles in erythroid and myeloid precursors

The patient received four units of packed red blood cells during the hospitalization. Her hemoglobin improved to 10.9 mg/dL, but she remained persistently mildly leukopenic and neutropenic. She had a history of intolerance to PO iron. The patient was started on IV iron sucrose infusions 100 mg/24 hours to treat her iron deficiency with plans to reattempt PO iron as an outpatient. Due to her persisting leukopenia, a bone marrow examination was requested after the second dose of IV iron sucrose. Bone marrow biopsy showed marrow cellularity of 65%‐75% with maturing trilineage hematopoiesis, including myeloid left shift. Rare ring sideroblasts (RSs) (Figure 1A) were detected on the iron stain, which were only <5% of erythroid precursors. No significant increase in blasts was seen, and there was no overt evidence of dysplasia, atypical lymphoid, or plasma cell population. Iron granules were present in plasma cells (Figure 1B). Hypercellular marrow (Figure 1C) and vacuoles of myeloid and erythroid precursors (Figure 1D) were seen on bone marrow examination. Flow cytometry was normal.

Due to the presence of RS with neutropenia, nonmalignant causes of sideroblastic anemia in our patient were reviewed. Nutritional derangements, such as zinc overload and copper deficiency, were considered. Interval history revealed that she was using denture adhesive zinc creams for years. Subsequently, her serum zinc level was found to be elevated (161 μg/dL, reference range: 60‐130), whereas the copper level was significantly low (<250 μg/L, reference range: 810‐1990).

She was recommended to stop using zinc creams, and 2 mg daily oral elemental copper was initiated. She was able to tolerate PO iron at low doses (ferrous sulfate 325 mg PO three times a day). After two months, the patient's anemia (Hb 12.4 g/dL), leukopenia (WBC count: 6.6 × 10^−3^/μL), and neutropenia (absolute neutrophils count: 3.9 × 10^3^/μL) were resolved. Her serum copper level increased from <250 to 614 μg/L, range: 810‐1990.

## 3 DISCUSSION

Dental denture creams are reported to cause hypocupremia, relatively more often than other causes of zinc excess; this is perhaps because of their increased absorption or higher zinc concentrations. According to SP Nations et al,^2^ zinc concentration ranges between 17 000 and 34 000 μg in each gram of commonly available denture creams. Zinc concentration in the serum correlates quantitatively with the amount of zinc applied. Interruption of zinc products leads to a rapid decline in serum zinc level, whereas their persistent use fosters persistent hyperzincemia.^2^ According to the World Health Organization expert consultation on vitamins and minerals requirements in humans, the upper limit of zinc intake in an adult is 45 mg/d; zinc intake higher than 50 mg/d starts disturbing copper homeostasis.^12^

Both copper and zinc are absorbed from the small intestine. Excessive presence of zinc in the body stimulates the production of metallothionein, a copper and zinc‐binding ligand, in enterocytes. Metallothionein proteins are stimulated by the zinc to curtail zinc toxicity by providing zinc‐binding sites. Metallothionein proteins, on the other hand, have a higher affinity for the copper than for the zinc. Due to increased metallothionein concentration in enterocytes, copper preferentially binds with the metallothionein, hampering its absorption into the systematic circulation, causing a copper‐depleted state. As the copper remains tethered inside the enterocytes, when enterocytes get sloughed off, the stored metallothionein‐copper complex also gets extruded in the feces.^13^

Sideroblastic anemia and neutropenia are one of the main hematologic features of hypocupremia, which can occur via various mechanisms.^1^ Copper depletion can cause (a) reduced absorption of iron from the intestines, (b) decreased release of iron from the reticuloendothelial system, (c) ineffective erythropoiesis due to diminished activity of copper‐containing enzymes, (d) increased destructions of erythrocytes, (e) ineffective myelopoiesis, (f) reduced number of progenitor cells, (g) increased destruction of copper‐depleted neutrophils, and (h) increased clearance of such neutrophils and development of anti‐neutrophilic antibodies.^14^, ^15^ In addition to nutritional disorders, neoplastic disorders such as MDS, toxins, or drugs, and some inherited diseases can cause sideroblastic anemia (Table 1: Major Causes of Sideroblastic Anemia).

Therefore, most of the time, hypocupremia‐related hematologic findings remain undetected and get labeled as other hematological disorders such as MDS. Cases of sideroblastic anemia with neutropenia due to the zinc‐induced copper deficiency are summarized in Table 2.

**TABLE 2:** Zinc‐induced hypocupremia, sideroblastic anemia, and neutropenia

Author, year	Age, gender	Hematologic presentation	Bone marrow findings	Cause of zinc excess/copper deficiency	Treatment
Porea et al, 2000^3^	17‐YO‐M	Normocytic anemia, neutropenia, and leukopenia	Cytoplasmic vacuolization of RBC precursors	Zinc‐containing acne cream	Discontinuation of zinc creams. No copper supplementation
Willis et al, 2005^4^	21‐YO‐M	Normocytic anemia, neutropenia, and leukopenia	Vacuolization of early granulocytes (myelocytes and promyelocytes), left shift maturation, dyserythropoiesis. RS‐4%.	Zinc cream for Dermatitis enteropathica	Discontinuation of zinc creams
Willis et al, 2005^4^	42‐YO‐M	Normocytic anemia, neutropenia, and leukopenia	As above. RS‐2%	Zinc‐related denture adhesive cream	Discontinuation of zinc Supplementation of copper
Khimani et al, 2013^5^	34‐YO‐M	Anemia, neutropenia, and leukopenia	Vacuolated erythroid and granulocyte precursors, Numerous RS	Denture adhesive cream	Discontinuation of Zinc. Oral copper 3 mg qd
Almeida et al, 2019^6^	59‐YO‐F	Anemia and leukopenia	Not done	Ingestion of metallic objects, Pica	Treatment of psychiatric disorder. Oral copper 2 mg qd
Cathcart et al, 2017^7^		Macrocytic anemia and neutropenia	Hypocellularity with mild dyspoietic features in the erythroid and granulocytic lineages	Denture adhesives	
Merza et al, 2015^8^	45‐YO‐F	Macrocytic anemia and neutropenia	Hypercellular with erythroid hyperplasia Cytoplasmic vacuolization of immature erythroid and granulocytic precursors	Possible multivitamins	Oral copper 2 mg TID
Atiq et al, 2018^10^	27‐YO‐M	Anemia, leukopenia, neutropenia, and rare dacrocytes	Not performed	Zinc‐related wound creams	Discontinuation of zinc Oral copper
Forman et al, 1990^26^	34‐YO‐M	Severe anemia	Myeloid hyperplasia, vacuolization in myeloid and erythroid precursors. 15% RS	Excess of zinc supplements	IV copper 2 mg q12 hourly for two weeks followed by copper 2 mg po daily
Wahab et al, 2019	30‐YO‐F	Normocytic anemia and neutropenia	Hypercellular marrow, iron granules in plasma cells, RS, vacuoles in erythroid and myeloid precursors	Excess oral ingestion of zinc in the form of zinc cream	Discontinuation of zinc cream. Oral copper and iron

Anemia with leukopenia is the most common hematologic finding of copper depletion, followed by isolated anemia and pancytopenia.^1^ Isolated thrombocytopenia and neutropenia are uncommon. Anemia could be microcytic, normocytic, and macrocytic. Normocytic anemia is more common than macrocytic anemia while microcytic anemia is least reported.^3^, ^4^, ^7^, ^8^ Bone marrow biopsy shows hypocellularity, erythroid hyperplasia, granulocytic hypoplasia, vacuolization of erythroid and myeloid precursors, excessive stainable iron in plasma cells and macrophages and RS.^1^ Vacuolization of myelocytes and promyelocytes is common in hypocupremia.^4^, ^5^, ^8^ Dyserythropoietic and dysmyelopoietic changes are common.^4^ Mild megaloblastic changes may also occur.^4^

RSs are not always present and could be as low as <1%.^1^ Therefore, the absence of RS does not exclusively rule out a copper deficiency. Hypercellularity of bone marrow has also been reported in case reports.^8^

Suspected hypocupremia‐related anemia should be evaluated with further testing such as serum copper, serum zinc, serum ceruloplasmin, and 24‐hour urinary copper excretion. In many cases of zinc‐induced hypocupremia, it is difficult to establish a source of zinc excess. Among 40 patients diagnosed with copper deficiency in one study, 16 patients had elevated zinc levels, but the source of zinc excess could be ascertained in only one patient.^1^ Other than hematologic findings, neurologic findings such as sensorimotor or cognitive deficits can also present concomitantly in a few cases.^2^ Copper supplementation, through oral or IV route, is the mainstay of the treatment. Zinc‐related copper deficiency might respond to the discontinuation of zinc supplements.^3^, ^4^ Oral copper is effective if gastrointestinal absorption is adequate, and the patient can tolerate oral formulation. Dosing regimens are chosen empirically and can be modified according to the hematologic and clinical response. One author used IV copper chloride or copper sulfate 1‐3 mg/d for 4‐5 days, followed by weekly administration, spanning over six months or more.^11^

It is important to remember copper deficiency as a potential etiology in patients presenting with cytopenias, especially leukopenia and anemia. Earlier detection avoids future morbidity of neurological deficits, a decline in quality of life due to cytopenias, and avoids continued ineffective interventions. This is especially important in patients who are considered to have myelodysplastic syndromes and are started on erythropoiesis‐stimulating agents and/or granulocyte colony‐stimulating factors and may be referred for allogeneic bone marrow transplantation.^16^ Once identified correctly and offending agents are removed, treatment is very effective with the reversal of cytopenias.

## CONFLICT OF INTEREST

This manuscript is original research, has not been previously published, and has not been submitted for publication elsewhere while under consideration. The authors declare no conflict of interest with this manuscript.

Consent: Consent was obtained for the publication of this case.

## AUTHOR CONTRIBUTION

AW: designed the study, wrote the case section, and also wrote the introduction and part of discussion. KM: did a literature search and summarized the data in tables. SB: provided the pathology images and edited the pathology section. NB: managed the patient, provided a hematology consultation, and wrote hematology part of the discussion. All authors contributed to the final editing and revision of the manuscript.
